# Blood pressure, hypertension, and the risk of aortic aneurysm in the UK Biobank

**DOI:** 10.1186/s12872-025-05279-2

**Published:** 2025-11-10

**Authors:** Rida E.Z. Naqvi, Ghaliah Baroom, Lili Zheng, Makoto Hibino, Antonio Berlanga-Taylor, Alicia K. Heath, Dagfinn Aune

**Affiliations:** 1 Department of Epidemiology and Biostatistics, School of Public Health, Imperial College London, White City Campus, 90 Wood Lane, London, W12 0BZ UK; 2 Department of Thoracic and Cardiovascular Surgery, Heart, Vascular, and Thoracic Institute, Cleveland Clinic, Cleveland, OH USA; 3 Unidad de Medicina de Datos, Centro Médico Nacional Siglo XXI, Instituto Mexicano del Seguro Social, Mexico City, Mexico; 4 Department of Nutrition, Oslo New University College, Oslo, Norway; 5 Department of Research, Cancer Registry of Norway, Norwegian Institute of Public Health, Oslo, Norway

**Keywords:** Blood pressure, Hypertension, Aortic aneurysm, Prospective cohort, UK biobank

## Abstract

**Background:**

Although an association between elevated blood pressure and risk of aortic aneurysm is established, few studies have investigated the association with aortic aneurysm subtypes. We investigated the association between systolic and diastolic blood pressure and hypertension status with the risk of aortic aneurysm in the UK Biobank.

**Methods:**

The analysis included 495,542 men and women aged 37–73 years at recruitment between 2006 and 2010. Aortic aneurysm cases were identified by linkage to hospitalization and mortality records. Multivariable Cox proportional hazards regression was used to estimate hazard ratios (HRs) and 95% confidence intervals (CIs) for the association between blood pressure and hypertension and risk of aortic aneurysm overall and for subtypes.

**Results:**

During a mean follow-up of 12.3 years, 3,346 incident aortic aneurysm cases were identified. Hypertension vs. no hypertension was associated with increased risk (HR, 95% CI) of aortic aneurysm (1.17, 1.08–1.27), and for thoracic (1.23, 1.04–1.46), abdominal (1.16, 1.04–1.30), and non-ruptured (1.18, 1.08–1.28) aortic aneurysm, and suggestively with unspecified site aortic aneurysm (1.18, 0.96–1.46) and aortic aneurysm mortality (1.26, 0.87–1.82), but not ruptured aortic aneurysm (1.02, 0.67–1.58). Systolic blood pressure was not associated with risk of aortic aneurysm overall or for any subtype. Diastolic blood pressure was positively associated with aortic aneurysm (1.74, 1.26–2.41, p_trend_<0.0001) for ≥ 110 vs. <80 mmHg, abdominal aortic aneurysm (1.95, 1.28–2.96, p_trend_<0.0001), unspecified site aortic aneurysm (2.02, 0.94–4.33, p_trend_=0.005), non-ruptured aortic aneurysm (1.79, 1.29–2.47), and aortic aneurysm mortality (2.32, 0.56–9.58, p_trend_<0.0001), and with ruptured aortic aneurysm (2.48, 1.22–5.03, p_trend_<0.0001 for 100–109 vs. <80 mmHg), while the association with thoracic aortic aneurysm was less clear (1.30, 0.64–2.63). These associations were strengthened and positive associations emerged for systolic blood pressure and abdominal and non-ruptured aortic aneurysm in sensitivity analyses when excluding participants with prevalent ischemic heart disease, stroke, those using hypertension medications and the first 3 years of follow-up.

**Conclusion:**

We found that hypertension status and higher diastolic blood pressure were associated with increased risk of aortic aneurysm overall and most aortic aneurysm subtypes. No association was observed for systolic blood pressure. Although further studies are needed on aortic aneurysm subtypes, these findings provide strong support that controlling blood pressure is important for reducing the risk of aortic aneurysm.

**Supplementary Information:**

The online version contains supplementary material available at 10.1186/s12872-025-05279-2.

## Introduction

Aortic aneurysm is defined as a dilation of the aortic wall by 1.5 times its normal diameter or ≥ 3 cm [1]. Aortic aneurysms can rupture, often resulting in high mortality [2]. The two main types of aortic aneurysm are thoracic and abdominal aortic aneurysms. Abdominal aortic aneurysm is a cause of around 175,000 deaths worldwide annually [3]. Previous studies have reported that higher age [4, 5], male sex [5–7], connective tissue diseases such as Marfan syndrome and Ehlers-Danlos syndrome [8], smoking [9], elevated total and low-density lipoprotein (LDL) cholesterol (4;6), and high body mass index (BMI) [10–13] are risk factors for aortic aneurysm, while physical activity [14], higher HDL cholesterol [4] and diabetes [5, 13, 15] may be associated with reduced risk.

Elevated blood pressure or hypertension is a major cardiovascular risk factor and has been associated with increased risk of a range of cardiovascular diseases [16–21], including aortic aneurysm [16, 22]. Several previous studies have suggested hypertension or elevated systolic and diastolic blood pressure is associated with increased risk of aortic aneurysm, although not all studies were fully consistent. However, a meta-analysis confirmed an increased risk of abdominal aortic aneurysm of 66% for persons with hypertension compared to those without hypertension, and both elevated systolic and diastolic blood pressure was associated with increased risk with summary relative risks (95% confidence intervals, CIs) of 1.14 (1.06–1.23) per 20 mmHg of systolic blood pressure and 1.28 (1.12–1.46) per 10 mmHg of diastolic blood pressure [22]. Previous studies have investigated the association between blood pressure or hypertension and risk of abdominal aortic aneurysm or any aortic aneurysm overall [22], but few have investigated aortic aneurysm subtypes [23]. Further studies are therefore needed to clarify whether the association between blood pressure and hypertension and aortic aneurysm differs for different subtypes (e.g. thoracic, abdominal, unspecified) or by ruptured or not ruptured status. We therefore investigated the association between blood pressure and hypertension and the risk of aortic aneurysm overall and by subtypes in the UK Biobank study, a large cohort study of 0.5 million British adults to further explore these associations.

## Methods

### Study population

The study population comprised participants from UK Biobank, a prospective cohort study of 502,656 UK residents including men and women mostly between 40 and 69 years of age and recruited between 2006 and 2010 [24]. The aim of this study is to identify causative factors of various chronic illnesses, which may advance prevention, diagnosis, treatment and management of chronic diseases. National Information Governance Board for Health and Social Care -England and Wales, Community Health Index Advisory Group in Scotland and North-West Multi-Centre Research Ethics Committee provided the ethical approval for the UK Biobank study. A central recruitment strategy was adopted, and individuals residing within 25 miles distance from 22 NHS assessment centres were invited to participate. Participation was voluntary and written informed consent was obtained. Participants attended a baseline assessment center where physical measurements were taken and biological samples like blood, urine and saliva were collected. Touchscreen questionnaires were completed at the baseline assessment center, including questions about sociodemographic profile, qualification, occupation, ethnicity, lifestyle, diet, medical history and sex-specific questions.

The dataset for the current project included 502,359 participants. We excluded in subsequent order persons with prevalent aortic aneurysm at baseline (either self-reported or with hospitalization record) (*n* = 476), different self-reported vs. genetic sex (*n* = 372), missing data on BMI (*n* = 3,096), smoking status (*n* = 2,422), and systolic blood pressure (*n* = 451), leaving 495,542 participants for inclusion in the analysis of blood pressure or hypertension and risk of aortic aneurysm.

### Assessment of exposure

Blood pressure was measured using OmronHEM-7015IT digital blood pressure monitors. The monitor automatically downloads and sends the blood pressure readings to the IT system of UK Biobank. This procedure was repeated twice with one-minute rest break to take an average to take into account blood pressure-fluctuations.

We categorized hypertension as a dichotomous variable based on direct measurements of blood pressure (with the cut-off at ≥ 140/≥90 vs. <140/<90 mmHg), and in addition we included self-reported diagnoses of hypertension and hospital records to define participants with hypertension (ICD-10 code I10, and ICD-9 code 401) at baseline. The association between systolic and diastolic blood pressure and aortic aneurysm risk was explored categorically, with cut-off values for systolic blood pressure of < 120, 120-<130, 130-<140, 140-<160, 160-<180, ≥ 180 mmHg, and for diastolic blood pressure of < 80, 80-<85, 85-<90, 90-<99, 100–109, ≥ 110 mmHg) and continuously per 20 and 10 mmHg, respectively, consistent with our previous publication [17].

### Assessment of outcome

Incident aortic aneurysm cases were identified through record linkage to Hospital Episode Statistics data and death registry data from the NHS. The diagnosis of aortic aneurysm was defined according to the 10th revision of the International Statistical Classification of Diseases and Related Health Problems (ICD-10) codes I71.1-I71.9 and ICD-9 codes 441.1–441.9.1.9 (ICD-9 codes were only used to define prevalent cases). Aortic aneurysm subtypes were classified as follows: thoracic aortic aneurysm (I71.1-I71.2), abdominal aortic aneurysm (I71.3-I71.4), thoracoabdominal aortic aneurysm (I71.5-I71.6), and unspecified site aortic aneurysm (I71.8-I71.9), ruptured aortic aneurysm (I71.1, I71.3, I71.5, I71.8), and non-ruptured aortic aneurysm (I71.2, I71.4, I71.6, I71.9). Follow-up of study participants began at the date of recruitment and for aortic aneurysm incidence ended at the date of aortic aneurysm diagnosis, death, loss to follow-up or the date of last complete follow-up (30 September 2021 for England and Wales and 31 October 2021 for Scotland), while for aortic aneurysm mortality participants were censored at the time of aortic aneurysm death, other causes of death, loss to follow-up, or the date of last complete follow-up (30 September 2021 for England and Wales and 31 October 2021 for Scotland).

### Statistical analysis

Multivariable Cox proportional hazards models were used to estimate hazard ratios (HRs) and 95% confidence intervals (CIs) for the association between hypertension, systolic blood pressure and diastolic blood pressure and risk of aortic aneurysm and its subsites with time since baseline as the underlying time metric. There was no violation of the proportional hazards assumption using Schoenfeld’s test (*p* = 0.37 for hypertension). For systolic and diastolic blood pressure analyses were conducted both for categories of blood pressure and on a continuous scale per 20 and 10 mmHg, respectively. Tests for linear trends were conducted by replacing each blood pressure category number with the median value within each category and entering this variable as a continuous variable in the models.

Based on prior subject knowledge the main multivariable analyses included adjustment for age, sex, ethnicity (Caucasian or non-Caucasian), education (college or university degree, other professional qualifications, national examination at ages 17–18 years, national examination at age 16 years, missing), a smoking variable combining smoking status, time since quitting smoking, and number of cigarettes per day (never, former & quit for ≥ 30 years, former & quit for 20-<30 years, former & quit for 10-<20 years, former & quit for < 10 years, former & missing years since quitting, current & <10 cigarettes/day, current & 10-<20 cigarettes/day, current & ≥20 cigarettes/day, current & missing number of cigarettes/day), alcohol consumption (never, special occasions only, 1–3 times/month, 1–2 times/week, 3–4 times/week, almost daily-daily), height (sex-specific quintiles), body mass index (in kg/m^2^, < 18.5, 18.5–<25.0, 25.0–<30.0, 30.0–<35.0, ≥ 35.0), leisure-time physical activity (quintiles, missing), connective tissue disease (Marfan’s syndrome, Ehlers-Danlos syndrome) (yes vs. no), low-density lipoprotein cholesterol (quintiles, missing), use of lipid-lowering medications (yes. vs. no), diabetes (yes vs. no), and use of hypertensive medications (yes vs. no). Hypertensive medications included the following drug classes: diuretics, calcium channel blockers, alpha-1 blockers, alpha-2 blockers, beta blockers, combined alpha and beta blockers, antihypertensives, combination drugs, aldosterone receptor blockers, angiotensin-II-receptor blockers, angiotensin converting enzyme inhibitors, and vasodilators. Sensitivity analyses were conducted to reduce potential impact of reverse causation on the results by excluding persons with ischemic heart disease or stroke at baseline, excluding persons using medications for hypertension, and by excluding the first three years of follow-up.

Statistical tests used in the analysis were all two-sided, and a p-value of < 0.05 was considered statistically significant. All analyses were performed using Stata/IC version 16.0 (StataCorp, College Station, TX, USA).

## Results

The current analysis included 495,542 participants (225,551 men and 269,991 women) with a mean (median) age of 56.5 years (58 years). During a mean follow-up of 12.3 years and total follow-up of 6,087,712 person-years, 3,346 aortic aneurysm cases occurred, including 775 thoracic, 2,028 abdominal, 26 thoracoabdominal, and 517 unspecified site, 121 ruptured and 3,225 non-ruptured aortic aneurysm cases, and 184 aortic aneurysm deaths. The number of thoracoabdominal aortic aneurysm cases was too low for analyses to be conducted.

A higher proportion of men than women and whites than non-whites had hypertension (Table 1). In addition, participants with hypertension (*n* = 269,419), when compared to those without hypertension, were on average older, had a lower education, were less likely to be current smokers, but more likely to be former smokers, were more likely to drink alcohol almost daily or daily, had a higher BMI and were more likely to be overweight and obese, were slightly shorter, had a slightly higher LDL-cholesterol, and higher prevalence of lipid-lowering medication use, diabetes mellitus, and use of medications for hypertension. No differences were observed in frequency of leisure-time physical activity and the prevalence of connective tissue diseases (Table 1).

**Table 1:** Baseline characteristics of participants according to hypertension status

Characteristics		No hypertension	Hypertension
N		226,123	269,419
Age, median		54	60
Sex (%)	Men	38.4	51.5
	Women	61.6	48.5
Ethnicity (%)	White	94.1	94.6
	Non-white	5.6	5.1
	Missing	0.3	0.3
Education (%)	Highest level	37.5	27.8
Smoking status (%)	Never	57.1	52.8
	Former	31.3	37.5
	Current	11.6	9.7
Alcohol Intake, (%)	Never	7.9	8.1
	Special occasions only	11.4	11.7
	1–3 times/month	12.1	10.4
	1–2 times/week	27.1	24.8
	3–4 times/week	23.4	22.8
	Daily/almost daily	18.1	22.2
	Missing	0.1	0.1
Leisure-time physical activity, frequency/week (median)		11	11
BMI, kg/m^2^ (median)		25.5	27.8
BMI categories, kg/m^2^	< 18.5	0.8	0.3
	18.5–24.9	43.5	23.4
	25–29.9.9	40.3	44.4
	30–34.5.5	12.0	22.2
	≥ 35	3.4	9.8
Height, men/women, cm (median)		176.5/163.2	175.1/161.6
Connective tissue disease (%)		0.05	0.04
LDL-cholesterol, mmol/L (median)		3.50	3.54
Lipid-lowering medication use (%)		8.7	25.7
Diabetes mellitus (%)		2.5	7.7
Hypertension medication use (%)		2.9	29.8

### Hypertension and risk of aortic aneurysm

Hypertension vs. no hypertension was positively associated with risk (HR, 95% CI) of aortic aneurysm (1.17, 1.08–1.27), thoracic aortic aneurysm (1.23, 1.04–1.46), abdominal aortic aneurysm (1.16, 1.04–1.30), and non-ruptured aortic aneurysm (1.18, 1.08–1.28), and suggestively with unspecified aortic aneurysm (1.18, 0.96–1.46) and aortic aneurysm mortality (1.26, 0.87–1.82), but not with ruptured aortic aneurysm (1.02, 0.67–1.58) (Table 2). These associations were strengthened in sensitivity analyses excluding participants with ischemic heart disease, stroke, users of hypertension medications and the first three years of follow-up, with HRs of 1.26 (1.13–1.40) for aortic aneurysm, 1.25 (1.02–1.52) for thoracic aortic aneurysm, 1.28 (1.11–1.48) for abdominal aortic aneurysm, 1.26 (1.13–1.41) for non-ruptured aortic aneurysm, 1.26 (0.98–1.63) for unspecified aortic aneurysm, and 1.59 (0.94–2.68) for aortic aneurysm mortality, but the association with ruptured aortic aneurysm remained null (1.08, 0.61–1.91) (Supplementary Table 1).

**Table 2:** Hazard ratios and 95% confidence intervals for the association between hypertension and subtypes of aortic aneurysm, ruptured vs. non-ruptured aortic aneurysm, and aortic aneurysm mortality

Outcome	Participants	No hypertension	Hypertension
		226,123	269,419
	Person-years	2,803,567.6	3,284,144.6
Aortic aneurysm	Cases	843	2,503
	HR (95% CI)	1.00	1.17 (1.08–1.27)
Thoracic aortic aneurysm	Cases	226	549
	HR (95% CI)	1.00	1.23 (1.04–1.46)
Abdominal aortic aneurysm	Cases	467	1561
	HR (95% CI)	1.00	1.16 (1.04–1.30)
Unspecified aortic aneurysm	Cases	144	373
	HR (95% CI)	1.00	1.18 (0.96–1.46)
Ruptured aortic aneurysm	Cases	32	89
	HR (95% CI)	1.00	1.02 (0.67–1.58)
Non-ruptured aortic aneurysm	Cases	811	2414
	HR (95% CI)	1.00	1.18 (1.08–1.28)
Aortic aneurysm mortality	Cases	41	143
	HR (95% CI)	1.00	1.26 (0.87–1.82)

Multivariable model adjusted for: age, sex, ethnicity, education, smoking status/time since quitting smoking/cigarettes per day, alcohol, height, body mass index, leisure-time physical activity, connective tissue disease (Marfan syndrome, Ehlers-Danlos syndrome), diabetes mellitus, low-density-lipoprotein cholesterol, lipid-lowering medications, blood pressure-lowering medications

### Systolic blood pressure and aortic aneurysm

No clear association was observed between higher systolic blood pressure and aortic aneurysm (0.95, 0.77–1.18), or for thoracic (0.95, 0.61–1.48), abdominal (0.96, 0.73–1.25), unspecified site (0.94, 0.53–1.66), ruptured (0.32, 0.07–1.43), non-ruptured (0.98, 0.79–1.22) aortic aneurysm, or for aortic aneurysm mortality (1.30, 0.62–2.76) when comparing ≥ 180 vs. <120 mmHg (Table 3). However, in sensitivity analyses when excluding participants with ischemic heart disease, stroke, users of hypertension medications and the first three years of follow-up, slight positive associations emerged for systolic blood pressure and aortic aneurysm overall (1.06, 1.00–1.12), abdominal aortic aneurysm (1.09, 1.01–1.17), and non-ruptured aortic aneurysm (1.06, 1.01–1.13) per 20 mmHg increase in systolic blood pressure (Supplementary Table 2).

**Table 3:** Hazard ratios and 95% confidence intervals for the association between systolic blood pressure and aortic aneurysm overall, thoracic, abdominal and unspecified aortic aneurysm, ruptured vs. non-ruptured aortic aneurysm, and aortic aneurysm mortality

Systolic blood pressure, cut-offs (median)									
Outcome		<120 (113.5)	120-<130 (125)	130-<140 (134.5)	140 < 160 (144.5)	>160-<179 (160)	≥ 180	P_trend_	Per 20 mmHg
	Participants	80,930	96,234	107,282	150,156	49,813	11,127		495,542
	Person-years	1,001,579.2	1,188,903.8	1,320,666.8	1,837,715.8	604,963.6	133,882.9		6,087,712
Aortic aneurysm	Cases	329	524	654	1,247	470	122		3,346
	HR (95% CI)	1.00	0.95 (0.82–1.08)	0.82 (0.72–0.94)	0.87 (0.77–0.98)	0.86 (0.75–1.00)	0.95 (0.77–1.18)	0.23	0.98 (0.94–1.02)
Thoracic aortic aneurysm	Cases	93	129	155	280	92	26		775
	HR (95% CI)	1.00	0.89 (0.68–1.16)	0.78 (0.60–1.02)	0.84 (0.66–1.07)	0.76 (0.56–1.02)	0.95 (0.61–1.48)	0.28	0.95 (0.87–1.03)
Abdominal aortic aneurysm	Cases	180	305	375	781	310	77		2,028
	HR (95% CI)	1.00	0.98 (0.82–1.18)	0.81 (0.68–0.97)	0.91 (0.77–1.07)	0.94 (0.78–1.13)	0.96 (0.73–1.25)	0.88	1.00 (0.95–1.05)
Unspecified aortic aneurysm	Cases	52	88	120	175	66	16		517
	HR (95% CI)	1.00	1.04 (0.73–1.46)	1.01 (0.73–1.41)	0.86 (0.62–1.18)	0.88 (0.60–1.28)	0.94 (0.53–1.66)	0.21	0.94 (0.85–1.04)
Ruptured aortic aneurysm	Cases	13	17	26	45	18	2		121
	HR (95% CI)	1.00	0.77 (0.37–1.59)	0.80 (0.41–1.56)	0.73 (0.39–1.36)	0.72 (0.35–1.49)	0.32 (0.07–1.43)	0.18	0.87 (0.71–1.07)
Non-ruptured aortic aneurysm	Cases	316	507	628	1,202	452	120		3,225
	HR (95% CI)	1.00	0.95 (0.83–1.10)	0.82 (0.72–0.94)	0.87 (0.77–0.99)	0.87 (0.75–1.01)	0.98 (0.79–1.22)	0.33	0.98 (0.94–1.02)
Aortic aneurysm mortality	Cases	20	23	35	66	29	11		184
	HR (95% CI)	1.00	0.70 (0.38–1.28)	0.73 (0.42–1.27)	0.74 (0.45–1.24)	0.85 (0.48–1.53)	1.30 (0.62–2.76)	0.46	1.05 (0.89–1.23)

Multivariable model adjusted for: age, sex, ethnicity, education, smoking status/time since quitting smoking/cigarettes per day, alcohol, height, body mass index, leisure-time physical activity, connective tissue disease (Marfan syndrome, Ehlers-Danlos syndrome), diabetes mellitus, low-density-lipoprotein cholesterol, lipid-lowering medications, blood pressure-lowering medications

### Diastolic blood pressure and aortic aneurysm

Higher diastolic blood pressure (≥ 110 vs. <80 mmHg) was positively associated with aortic aneurysm (1.74, 1.26–2.41, p_trend_<0.0001), abdominal (1.95, 1.28–2.96, p_trend_<0.0001), unspecified (2.02, 0.94–4.33, p_trend_=0.003), ruptured (2.48, 1.22–5.03, p_trend_=0.02 for 100–109 vs. <80 mmHg), and non-ruptured (1.79, 1.29–2.47, p_trend_<0.0001) aortic aneurysm, and aortic aneurysm mortality (2.32, 0.45–9.58, p_trend_<0.0001), but no association was observed for thoracic aortic aneurysm (1.30, 0.64–2.63, p_trend_=0.76) (Table 4). In sensitivity analyses excluding participants with ischemic heart disease, stroke, users of hypertension medications and the first three years of follow-up, the observed associations were in general strengthened, with HRs of 1.91 (1.24–2.94) for aortic aneurysm, 2.15 (1.23–3.77) for abdominal, 3.04 (1.22–7.58) for unspecified site, 2.07 (0.68–6.29) for ruptured, and 1.96 (1.27–3.02) for non-ruptured aortic aneurysm, and 2.75 (1.15–6.59 for 100–109 vs. <80 mmHg) for aortic aneurysm mortality (Supplementary Table 3).

**Table 4:** Hazard ratios and 95% confidence intervals for the association between diastolic blood pressure and aortic aneurysm overall, thoracic, abdominal and unspecified aortic aneurysm, ruptured vs. non-ruptured aortic aneurysm, and aortic aneurysm mortality

Diastolic blood pressure, cut-offs (median), mmHg									
Outcome		< 80 (74)	80–84 (82)	85–89 (87)	90–99 (93.5)	100–109 (103)	≥ 110 (113)	P_trend_	Per 10 mmHg
	Participants	206,226	96,709	81,526	87,506	20,248	3,327		495,542
	Person-years	2,530,245.2	1,188,968.1	1,002,830.9	1,076,816.9	248,468.5	40,382.6		6,087,712
Aortic aneurysm	Cases	1,213	606	541	744	204	38		3,346
	HR (95% CI)	1.00	0.95 (0.86–1.05)	0.95 (0.86–1.05)	1.19 (1.08–1.31)	1.43 (1.23–1.67)	1.74 (1.26–2.41)	< 0.0001	1.08 (1.04–1.12)
Thoracic aortic aneurysm	Cases	308	143	115	158	43	8		775
	HR (95% CI)	1.00	0.85 (0.70–1.04)	0.76 (0.61–0.94)	0.93 (0.76–1.13)	1.08 (0.78–1.50)	1.30 (0.64–2.63)	0.76	0.96 (0.89–1.04)
Abdominal aortic aneurysm	Cases	715	370	335	461	124	23		2,028
	HR (95% CI)	1.00	1.01 (0.89–1.15)	1.04 (0.91–1.18)	1.31 (1.17–1.48)	1.58 (1.30–1.92)	1.95 (1.28–2.96)	< 0.0001	1.13 (1.08–1.18)
Unspecified aortic aneurysm	Cases	181	86	89	117	37	7		517
	HR (95% CI)	1.00	0.88 (0.68–1.15)	1.02 (0.79–1.33)	1.21 (0.95–1.54)	1.67 (1.16–2.40)	2.02 (0.94–4.33)	0.003	1.14 (1.04–1.24)
Ruptured aortic aneurysm	Cases	41	25	12	33	10	0		121
	HR (95% CI)	1.00	1.24 (0.75–2.05)	0.69 (0.36–1.32)	1.82 (1.14–2.92)	2.48 (1.22–5.03)	-	0.02	1.23 (1.03–1.47)
Non-ruptured aortic aneurysm	Cases	1,172	581	529	711	194	38		3,225
	HR (95% CI)	1.00	0.94 (0.85–1.04)	0.96 (0.86–1.06)	1.17 (1.06–1.29)	1.40 (1.20–1.64)	1.79 (1.29–2.47)	< 0.0001	1.08 (1.04–1.11)
Aortic aneurysm mortality	Cases	58	28	34	51	11	2		184
	HR (95% CI)	1.00	1.01 (0.64–1.60)	1.40 (0.91–2.16)	1.99 (1.35–2.93)	1.93 (1.00–3.72)	2.32 (0.56–9.58)	< 0.0001	1.25 (1.09–1.45)

Multivariable model adjusted for: age, sex, ethnicity, education, smoking status/time since quitting smoking/cigarettes per day, alcohol, height, body mass index, leisure-time physical activity, connective tissue disease (Marfan syndrome, Ehlers-Danlos syndrome), diabetes mellitus, low-density-lipoprotein cholesterol, lipid-lowering medications, blood pressure-lowering medications

## Discussion

In this large-scale analysis from the UK Biobank a history of hypertension or elevated diastolic blood pressure was associated with increased risk of aortic aneurysm overall as well as for most of the subtypes of aortic aneurysm, including thoracic, abdominal, unspecified site, non-ruptured aortic aneurysms, and suggestively for aortic aneurysm mortality. No association was observed for ruptured aortic aneurysm, and the association with thoracic aortic aneurysm was only observed for hypertension status, and not for diastolic blood pressure. No association was in general observed between systolic blood pressure and any aortic aneurysm outcome in the main analyses. In sensitivity analyses many of the positive associations between hypertension and diastolic blood pressure and aortic aneurysm risk were strengthened and additional positive associations emerged for systolic blood pressure and aortic aneurysm overall and for abdominal and non-ruptured aortic aneurysms.

Our finding of an increased risk of aortic aneurysm with hypertension is consistent with most previous studies [4–7, 25–36]. The results are also consistent with a meta-analysis of 21 cohort studies with 28,162 cases and 5,440,588 participants that reported an increased risk, although the current association is somewhat weaker than what was previously reported (16–28% increase in risk of abdominal aortic aneurysm in UK Biobank vs. a 66% increase in risk in the meta-analysis) [22]. It is possible that the weaker association in the current study is partly explained by a higher use of anti-hypertensive medications in this population than in earlier studies or that it could be due to random variation. However, little data is currently available on aortic aneurysm subsites or subtypes. A recent study from Sweden reported positive associations between hypertension and both abdominal aortic aneurysm (odds ratio 2.2, 95% CI: 1.4–3.3) and thoracic aortic aneurysm (1.7, 95% CI: 1.1–2.7) [23], which is directionally consistent with our current findings in UK Biobank.

Although we found little evidence of an association between higher systolic blood pressure and aortic aneurysm in the main analysis, we did observe an association in sensitivity analyses after excluding participants with ischemic heart disease, stroke, those using medications for hypertension and when excluding the first three years of follow-up. This could indicate that reverse causation may have been at play in the main analysis, as patients with ischemic heart disease and stroke often are treated with medications for hypertension. The finding from the sensitivity analysis is consistent with a meta-analysis which reported a summary relative risk of 1.14 (1.06–1.23) for abdominal aortic aneurysm per 20 mmHg increase in systolic blood pressure [22], whilst our analysis showed a HR of 1.09 (1.01–1.17) per 20 mmHg, and these findings are also directionally consistent with additional studies [4, 35, 36] that have been published since the meta-analysis.

We found a strong positive association between higher diastolic blood pressure and risk of aortic aneurysm overall and for most subtypes with roughly 2-fold increases in risk at the highest level of diastolic blood pressure. This finding is directionally consistent with a meta-analysis of six cohort studies, which reported a 28% increase in risk of abdominal aortic aneurysm per 10 mmHg increase in diastolic blood pressure and a 3–4-fold increase in risk at 100–110 vs. 67 mmHg. More recent studies have also reported increased aortic aneurysm risk with higher diastolic blood pressure [4, 35, 36]. Of these, a large Japanese cohort of 3.3 million participants and 2,177 aortic aneurysm cases reported a HR of 1.15 (1.12–1.17) of aortic aneurysm incidence per 5 mmHg increase in diastolic blood pressure (equal to 1.32, 1.25–1.37 per 10 mmHg), while another Japanese cohort reported a HR of 1.29 (1.11–1.50) for aortic aneurysm mortality per 10.7 mmHg increase in diastolic blood pressure [4]. A Swedish cohort reported a HR of 1.39 (1.27–1.52) for aortic aneurysm incidence with high diastolic blood pressure [35]. Another Japanese study using computer-based-3D-reconstruction-technology and clinical data, found elevated diastolic blood pressure to be one of the main predictors of aortic aneurysm rupture [37], a finding consistent with the 2-fold increase in risk of ruptured aortic aneurysm in the current study. Our findings are also partly consistent with Mendelian randomization studies, showing increased risk of aortic aneurysm with elevated blood pressure [38, 39], providing further support for potential causal associations.

As for the underlying mechanism(s) that may explain the association between elevated blood pressure and aortic aneurysm, hypertension can increase the risk of aortic aneurysm through atherosclerosis, by increasing wall tension, leading to impaired repair processes and aneurysm formation [40]. Experimental studies have shown that hypertension induces changes in the intima as well as myointimal thickening in the arteries of experimental animals [41, 42]. Hypertension can lead to shifts in elastin and collagen fiber content within the aorta wall and smooth muscle cells, which are characteristic of aortic aneurysm cases [43]. Higher blood pressure increases wall stress which can result in aortic enlargement. Prospective studies found higher risk of aortic root dilatation with higher diastolic [44, 45] and systolic blood pressure [44], although not all studies were consistent [46], and other studies have reported ascending aorta dilatation with higher diastolic blood pressure [47–49] and systolic blood pressure [48, 49]. A greater aortic size index was associated with a 2.5-fold increased risk of abdominal aortic aneurysm in one cohort study [50]. Lastly, hypertension is also a risk factor for other cardiovascular diseases [51] that may increase the risk of developing aortic aneurysm, including coronary heart disease [5, 26, 28], stroke [5, 28] and peripheral vascular disease [26]. The stronger association observed between diastolic blood pressure and aortic aneurysm than for systolic blood pressure may be because high systolic blood pressure, and low diastolic blood pressure contribute to arterial stiffness, which is protective for aortic aneurysms [16].

Strengths of the current study include (1) the prospective design which avoids recall bias and reduces the potential for selection bias, (2) the large sample size, moderately long follow-up and large number of cases which allowed for investigation of associations with aortic aneurysm subtypes, (3) the comprehensive adjustment for a wide range of other confounding risk factors, and (4) multiple sensitivity analyses which strengthened the conclusions of the study. Although we cannot exclude the possibility of residual confounding, our findings persisted after adjustment for a wide range of risk factors and given the relatively strong association between diastolic blood pressure and aortic aneurysm, that finding seems less likely to be explained by residual confounding. We did not have information on aortic diameter in the current study, therefore we were not able to assess asymptomatic cases that did not lead to hospitalization or death. A previous meta-analysis reported a summary RR of 1.40 (1.20–1.63, *n* = 3) for studies on hypertension and ultrasound detected aortic aneurysm and 1.73 (1.53–1.96, *n* = 10) for studies on hypertension and aortic aneurysm detected by linkages to medical, hospital or death records [22], with no significant heterogeneity between subgroups (*p* = 0.11), which suggests that this limitation would not alter the overall conclusion of an adverse effect of hypertension on aortic aneurysm risk. We cannot rule out the possibility for misclassification of aortic aneurysm subtypes because of inaccuracies in hospital and death records. Since our outcome assessment was based on hospital and death records, the diagnosis date reflects the date of clinical detection rather than actual disease onset, which may have led to misclassification of the event timing. Although the UK Biobank is not representative of the general population, associations between a risk factor and a disease can still be established. One last limitation is that the UK Biobank consists mostly of Caucasians, potentially limiting the generalisability of the results, however, we think the current findings may be more broadly applicable given that similar results have been observed in other populations with different ethnic backgrounds [4, 22, 35, 36].

Our findings provide further evidence that controlling blood pressure is important for the prevention of aortic aneurysm and therefore the findings have important public health implications. The findings are also broadly consistent with previous studies showing an adverse effect of elevated blood pressure across a range of cardiovascular disease outcomes [16–22, 38] and therefore reinforce the importance of blood pressure control for primary prevention. Given the lack of previous studies looking at aortic aneurysm subtypes, further large cohort studies are needed to explore these associations.

## Conclusion

We found that hypertension and higher diastolic blood pressure were associated with increased risk of aortic aneurysm overall and most aortic aneurysm subtypes, although no association was observed for systolic blood pressure. While further studies are needed on blood pressure and the less investigated aortic aneurysm subtypes, these findings provide strong support that controlling blood pressure is important for reducing the risk of aortic aneurysm.

## Supplementary Information

Supplementary Material 1.

## Data Availability

Data from the UK Biobank is available from https://www.ukbiobank.ac.uk.
