# Supplementary material for: Blood pressure, hypertension, and the risk of aortic aneurysm in the UK Biobank
Source: BMC Cardiovasc Disord. 2025 Nov 10;25:799. doi: 10.1186/s12872-025-05279-2 (PMC12604414; doi:10.1186/s12872-025-05279-2)
Supplement: Supplementary file 1 — Supplementary Material 1. [file 12872_2025_5279_MOESM1_ESM.docx]

**Supplementary material for**

Naqvi REZ, Baroom G, Zheng L, Hibino M, Berlanga-Taylor A, Heath AK, Aune D. Blood pressure, hypertension, and the risk of aortic aneurysm in the UK Biobank study.

Supplementary Table 1: Hazard ratios and 95% confidence intervals for the association between hypertension and subtypes of aortic aneurysm, ruptured vs. non-ruptured aortic aneurysm, and aortic aneurysm mortality - sensitivity analyses excluding participants with prevalent ischemic heart disease, stroke, users of blood-pressure-lowering medications, and the first 3 years of follow-up.

| Outcome |  | No hypertension | Hypertension |
| --- | --- | --- | --- |
|  | Participants | 226,123 | 269,419 |
|  | Person-years | 2,803,567.6 | 3,284,144.6 |
| Aortic aneurysm | Cases | 843 | 2,503 |
|  | HR (95% CI)^1^ | 1.00 | 1.17 (1.08-1.27) |
|  | HR (95% CI)^2^ excl. CVD | 1.00 | 1.23 (1.11-1.35) |
|  | HR (95% CI)^3^ excl. HTN meds | 1.00 | 1.22 (1.11-1.34) |
|  | HR (95% CI)^4^ excl. CVD+HTN meds | 1.00 | 1.25 (1.13-1.38) |
|  | HR (95% CI)^5^ excl. CVD+HTN meds+ first 3 years | 1.00 | 1.26 (1.13-1.40) |
| Thoracic aortic aneurysm | Cases | 226 | 549 |
|  | HR (95% CI)^1^ | 1.00 | 1.23 (1.04-1.46) |
|  | HR (95% CI)^2^ excl. CVD | 1.00 | 1.26 (1.04-1.52) |
|  | HR (95% CI)^3^ excl. HTN meds | 1.00 | 1.30 (1.08-1.57) |
|  | HR (95% CI)^4^ excl. CVD+HTN meds | 1.00 | 1.29 (1.06-1.56) |
|  | HR (95% CI)^5^ excl. CVD+HTN meds+ first 3 years | 1.00 | 1.25 (1.02-1.52) |
| Abdominal aortic aneurysm | Cases | 467 | 1561 |
|  | HR (95% CI)^1^ | 1.00 | 1.16 (1.04-1.30) |
|  | HR (95% CI)^2^ excl. CVD | 1.00 | 1.25 (1.09-1.43) |
|  | HR (95% CI)^3^ excl. HTN meds | 1.00 | 1.23 (1.08-1.40) |
|  | HR (95% CI)^4^ excl. CVD+HTN meds | 1.00 | 1.26 (1.10-1.45) |
|  | HR (95% CI)^5^ excl. CVD+HTN meds+ first 3 years | 1.00 | 1.28 (1.11-1.48) |
| Unspecified aortic aneurysm | Cases | 144 | 373 |
|  | HR (95% CI)^1^ | 1.00 | 1.18 (0.96-1.46) |
|  | HR (95% CI)^2^ excl. CVD | 1.00 | 1.19 (0.94-1.51) |
|  | HR (95% CI)^3^ excl. HTN meds | 1.00 | 1.12 (0.89-1.41) |
|  | HR (95% CI)^4^ excl. CVD+HTN meds | 1.00 | 1.18 (0.93-1.51) |
|  | HR (95% CI)^5^ excl. CVD+HTN meds+ first 3 years | 1.00 | 1.26 (0.98-1.63) |
| Ruptured aortic aneurysm | Cases | 32 | 89 |
|  | HR (95% CI)^1^ | 1.00 | 1.02 (0.67-1.58) |
|  | HR (95% CI)^2^ excl. CVD | 1.00 | 0.87 (0.53-1.42) |
|  | HR (95% CI)^3^ excl. HTN meds | 1.00 | 1.17 (0.71-1.91) |
|  | HR (95% CI)^4^ excl. CVD+HTN meds | 1.00 | 1.04 (0.62-1.75) |
|  | HR (95% CI)^5^ excl. CVD+HTN meds+ first 3 years | 1.00 | 1.08 (0.61-1.91) |
| Non-ruptured aortic aneurysm | Cases | 811 | 2414 |
|  | HR (95% CI)^1^ | 1.00 | 1.18 (1.08-1.28) |
|  | HR (95% CI)^2^ excl. CVD | 1.00 | 1.24 (1.13-1.37) |
|  | HR (95% CI)^3^ excl. HTN meds | 1.00 | 1.22 (1.11-1.34) |
|  | HR (95% CI)^4^ excl. CVD+HTN meds | 1.00 | 1.25 (1.13-1.39) |
|  | HR (95% CI)^5^ excl. CVD+HTN meds+ first 3 years | 1.00 | 1.26 (1.13-1.41) |
| Aortic aneurysm mortality | Cases | 41 | 143 |
|  | HR (95% CI)^1^ | 1.00 | 1.26 (0.87-1.82) |
|  | HR (95% CI)^2^ excl. CVD | 1.00 | 1.40 (0.90-2.19) |
|  | HR (95% CI)^3^ excl. HTN meds | 1.00 | 1.44 (0.93-2.21) |
|  | HR (95% CI)^4^ excl. CVD+HTN meds | 1.00 | 1.54 (0.96-2.46) |
|  | HR (95% CI)^5^ excl. CVD+HTN meds+ first 3 years | 1.00 | 1.59 (0.94-2.68) |

Multivariable model adjusted for: age, sex, ethnicity, education, smoking status/time since quitting smoking/cigarettes per day, alcohol, height, body mass index, leisure-time physical activity, connective tissue disease (Marfan syndrome, Ehlers-Danlos syndrome), diabetes mellitus, low-density-lipoprotein cholesterol, lipid-lowering medications, blood pressure-lowering medications

^1^ main model

^2^ excluding persons with prevalent ischemic heart disease and stroke

^3^ excluding persons using blood-pressure lowering medications

^4^ excluding persons with prevalent ischemic heart disease and stroke and using blood-pressure lowering medications

^5^ excluding persons with prevalent ischemic heart disease and stroke and using blood-pressure lowering medications and the first 3 years of follow-up

Supplementary Table 2: Hazard ratios and 95% confidence intervals for the association between systolic blood pressure and aortic aneurysm overall, thoracic, abdominal and unspecified aortic aneurysm, ruptured vs. non-ruptured aortic aneurysm, and aortic aneurysm mortality - sensitivity analyses excluding participants with prevalent ischemic heart disease, stroke, users of blood-pressure-lowering medications, and the first 3 years of follow-up.

| Systolic blood pressure, cut-offs (median) | | | | | | | | | | | | | | | | |
| --- | --- | --- | --- | --- | --- | --- | --- | --- | --- | --- | --- | --- | --- | --- | --- | --- |
| Outcome | |  | | | <120 (113.5) | 120-<130 (125) | | | 130-<140 (134.5) | 140<160 (144.5) | >160-<179 (160) | ≥180 | P_trend_ | | Per 20 mmHg | |
|  |  | Participants | | | 80,930 | 96,234 | | | 107,282 | 150,156 | 49,813 | 11,127 |  | |  | |
|  |  | Person-years | | | 1,001,579.2 | 1,188,903.8 | | | 1,320,666.8 | 1,837,715.8 | 604,963.6 | 133,882.9 |  | | 6,087,712 | |
| Aortic aneurysm | | Cases | | | 329 | 524 | | | 654 | 1,247 | 470 | 122 |  | | 3,346 | |
|  |  | HR (95% CI)^1^ | | | 1.00 | 0.95 (0.82-1.08) | | | 0.82 (0.72-0.94) | 0.87 (0.77-0.98) | 0.86 (0.75-1.00) | 0.95 (0.77-1.18) | 0.23 | | 0.98 (0.94-1.02) | |
|  |  | HR (95% CI)^2^ | | | 1.00 | 0.95 (0.0-1.12) | | | 0.88 (0.75-1.03) | 0.93 (0.80-1.08) | 0.92 (0.77-1.09) | 1.08 (0.85-1.37) | 0.29 | | 1.01 (0.96-1.05) | |
|  |  | HR (95% CI)^3^ | | 1.00 | | 0.98 (0.82-1.17) | | 0.83 (0.70-0.99) | | 1.00 (0.84-1.17) | 1.02 (0.84-1.23) | 1.05 (0.79-1.38) | 0.23 | | 1.04 (0.99-1.09) | |
|  |  | HR (95% CI)^4^ | | 1.00 | | 0.99 (0.82-1.21) | | 0.90 (0.74-1.09) | | 1.05 (0.88-1.26) | 1.07 (0.87-1.32) | 1.14 (0.85-1.53) | 0.08 | | 1.05 (1.00-1.11) | |
|  |  | HR (95% CI)^5^ | | 1.00 | | 0.98 (0.80-1.20) | | 0.93 (0.76-1.13) | | 1.06 (0.88-1.28) | 1.12 (0.90-1.38) | 1.13 (0.83-1.54) | 0.05 | | 1.06 (1.00-1.12) | |
| Thoracic aortic aneurysm | Cases | 93 | | | 129 | 155 | | | 280 | 92 | 26 |  | 775 | |  |  |
|  | HR (95% CI)^1^ | | 1.00 | | 0.89 (0.68-1.16) | | 0.78 (0.60-1.02) | | 0.84 (0.66-1.07) | 0.76 (0.56-1.02) | 0.95 (0.61-1.48) | 0.28 | | 0.95 (0.87-1.03) | |  |
|  | HR (95% CI)^2^ | | 1.00 | | 0.94 (0.70-1.27) | | 0.82 (0.61-1.10) | | 0.92 (0.70-1.21) | 0.77 (0.55-1.07) | 1.01 (0.62-1.63) | 0.42 | | 0.95 (0.87-1.04) | |  |
|  | HR (95% CI)^3^ | | 1.00 | | 0.99 (0.72-1.37) | | 0.86 (0.62-1.18) | | 0.97 (0.72-1.32) | 0.92 (0.63-1.34) | 1.01 (0.57-1.80) | 0.91 | | 0.99 (0.89-1.10) | |  |
|  | HR (95% CI)^4^ | | 1.00 | | 0.97 (0.69-1.35) | | 0.87 (0.62-1.21) | | 1.01 (0.74-1.38) | 0.92 (0.62-1.36) | 1.01 (0.55-1.83) | 0.96 | | 1.00 (0.90-1.11) | |  |
|  | HR (95% CI)^5^ | | 1.00 | | 1.00 (0.71-1.41) | | 0.90 (0.64-1.26) | | 0.98 (0.71-1.36) | 0.94 (0.64-1.40) | 1.00 (0.54-1.85) | 0.91 | | 0.98 (0.88-1.09) | |  |
| Abdominal aortic aneurysm | Cases | 180 | | | 305 | 375 | | | 781 | 310 | 77 |  | 2,028 | |  |  |
|  | HR (95% CI)^1^ | | 1.00 | | 0.98 (0.82-1.18) | | 0.81 (0.68-0.97) | | 0.91 (0.77-1.07) | 0.94 (0.78-1.13) | 0.96 (0.73-1.25) | 0.88 | | 1.00 (0.95-1.05) | |  |
|  | HR (95% CI)^2^ | | 1.00 | | 1.01 (0.79-1.30) | | 0.91 (0.72-1.16) | | 1.01 (0.81-1.26) | 1.06 (0.83-1.35) | 1.12 (0.81-1.54) | 0.22 | | 1.04 (0.98-1.10) | |  |
|  | HR (95% CI)^3^ | | 1.00 | | 0.99 (0.76-1.28) | | 0.84 (0.65-1.08) | | 1.10 (0.87-1.38) | 1.13 (0.87-1.46) | 1.05 (0.73-1.52) | 0.06 | | 1.07 (1.00-1.14) | |  |
|  | HR (95% CI)^4^ | | 1.00 | | 1.07 (0.79-1.43) | | 0.98 (0.74-1.30) | | 1.22 (0.93-1.59) | 1.25 (0.93-1.68) | 1.21 (0.81-1.81) | 0.02 | | 1.09 (1.01-1.16) | |  |
|  | HR (95% CI)^5^ | | 1.00 | | 0.99 (0.73-1.36) | | 0.98 (0.73-1.32) | | 1.20 (0.91-1.58) | 1.26 (0.93-1.72) | 1.18 (0.77-1.80) | 0.02 | | 1.09 (1.01-1.17) | |  |
| Unspecified aortic aneurysm | Cases | 52 | | | 88 | 120 | | | 175 | 66 | 16 |  | 517 | |  |  |
|  | HR (95% CI)^1^ | | 1.00 | | 1.04 (0.73-1.46) | | 1.01 (0.73-1.41) | | 0.86(0.62-1.18) | 0.88 (0.60-1.28) | 0.94 (0.53-1.66) | 0.21 | | 0.94 (0.85-1.04) | |  |
|  | HR (95% CI)^2^ | | 1.00 | | 0.91 (0.62-1.36) | | 1.00 (0.69-1.45) | | 0.86 (0.60-1.23) | 0.84 (0.55-1.29) | 1.08 (0.60-1.96) | 0.57 | | 0.97 (0.86-1.08) | |  |
|  | HR (95% CI)^3^ | | 1.00 | | 1.09 (0.72-1.64) | | 0.90 (0.59-1.35) | | 0.89 (0.60-1.32) | 0.96 (0.61-1.53) | 1.09 (0.55-2.17) | 0.73 | | 1.00 (0.88-1.13) | |  |
|  | HR (95% CI)^4^ | | 1.00 | | 1.01 (0.65-1.56) | | 0.91 (0.59-1.40) | | 0.90 (0.60-1.36) | 0.98 (0.60-1.59) | 1.18 (0.58-2.38) | 0.95 | | 1.03 (0.90-1.17) | |  |
|  | HR (95% CI)^5^ | | 1.00 | | 1.05 (0.66-1.68) | | 0.97 (0.61-1.54) | | 1.00 (0.64-1.55) | 1.13 (0.68-1.89) | 1.28 (0.61-2.70) | 0.52 | | 1.07 (0.93-1.23) | |  |
| Ruptured aortic aneurysm | Cases | 13 | | | 17 | 26 | | | 45 | 18 | 2 |  | 121 | |  |  |
|  | HR (95% CI)^1^ | | 1.00 | | 0.77 (0.37-1.59) | | 0.80 (0.41-1.56) | | 0.73 (0.39-1.36) | 0.72 (0.35-1.49) | 0.32 (0.07-1.43) | 0.18 | | 0.87 (0.71-1.07) | |  |
|  | HR (95% CI)^2^ | | 1.00 | | 0.88 (0.40-1.94) | | 0.65 (0.29-1.41) | | 0.53 (0.25-1.11) | 0.65 (0.28-1.49) | 0.34 (0.07-1.59) | 0.10 | | 0.81 (0.64-1.03) | |  |
|  | HR (95% CI)^3^ | | 1.00 | | 0.68 (0.28-1.64) | | 0.57 (0.24-1.35) | | 0.61 (0.28-1.33) | 0.90 (0.38-2.12) | 0.24 (0.03-1.90) | 0.64 | | 0.95 (0.73-1.23) | |  |
|  | HR (95% CI)^4^ | | 1.00 | | 0.77 (0.31-1.93) | | 0.60 (0.24-1.47) | | 0.57 (0.25-1.32) | 0.81 (0.32-2.05) | 0.25 (0.03-2.05) | 0.37 | | 0.88 (0.66-1.16) | |  |
|  | HR (95% CI)^5^ | | 1.00 | | 0.71 (0.26-1.93) | | 0.63 (0.24-1.64) | | 0.56 (0.23-1.38) | 0.76 (0.28-2.09) | - | 0.23 | | 0.84 (0.62-1.14) | |  |
| Non-ruptured aortic aneurysm | Cases | 316 | | | 507 | 628 | | | 1,202 | 452 | 120 |  | 3,225 | |  |  |
|  | HR (95% CI)^1^ | | 1.00 | | 0.95 (0.83-1.10) | | 0.82 (0.72-0.94) | | 0.87 (0.77-0.99) | 0.87 (0.75-1.01) | 0.98 (0.79-1.22) | 0.33 | | 0.98 (0.94-1.02) | |  |
|  | HR (95% CI)^2^ | | 1.00 | | 0.95 (0.80-1.13) | | 0.89 (0.75-1.05) | | 0.95 (0.81-1.11) | 0.93 (0.78-1.11) | 1.13 (0.88-1.43) | 0.54 | | 1.01 (0.97-1.06) | |  |
|  | HR (95% CI)^3^ | | 1.00 | | 1.00 (0.83-1.20) | | 0.84 (0.70-1.01) | | 1.02 (0.86-1.20) | 1.02 (0.84-1.24) | 1.09 (0.82-1.45) | 0.19 | | 1.04 (0.99-1.09) | |  |
|  | HR (95% CI)^4^ | | 1.00 | | 1.00 (0.82-1.23) | | 0.92 (0.76-1.12) | | 1.08 (0.90-1.30) | 1.09 (0.88-1.34) | 1.20 (0.89-1.61) | 0.047 | | 1.06 (1.00-1.12) | |  |
|  | HR (95% CI)^5^ | | 1.00 | | 0.99 (0.81-1.23) | | 0.94 (0.77-1.15) | | 1.09 (0.90-1.32) | 1.13 (0.91-1.41) | 1.20 (0.88-1.64) | 0.03 | | 1.06 (1.01-1.13) | |  |
| Aortic aneurysm mortality | Cases | 20 | | | 23 | 35 | | | 66 | 29 | 11 |  | 184 | |  |  |
|  | HR (95% CI)^1^ | | 1.00 | | 0.70 (0.38-1.28) | | 0.73 (0.42-1.27) | | 0.74 (0.45-1.24) | 0.85 (0.48-1.53) | 1.30 (0.62-2.76) | 0.46 | | 1.05 (0.89-1.23) | |  |
|  | HR (95% CI)^2^ | | 1.00 | | 0.58 (0.28-1.17) | | 0.56 (0.29-1.08) | | 0.63 (0.35-1.14) | 0.73 (0.37-1.44) | 1.18 (0.50-2.76) | 0.46 | | 1.07 (0.89-1.29) | |  |
|  | HR (95% CI)^3^ | | 1.00 | | 0.62 (0.28-1.36) | | 0.61 (0.29-1.28) | | 0.79 (0.41--1.53) | 0.71 (0.32-1.57) | 1.34 (0.51-3.51) | 0.49 | | 1.08 (0.88-1.34) | |  |
|  | HR (95% CI)^4^ | | 1.00 | | 0.64 (0.27-1.51) | | 0.66 (0.30-1.47) | | 0.82 (0.39-1.69) | 0.74 (0.31-1.76) | 1.39 (0.49-3.95) | 0.49 | | 1.10 (0.88-1.38) | |  |
|  | HR (95% CI)^5^ | | 1.00 | | 0.58 (0.23-1.47) | | 0.50 (0.21-1.23) | | 0.83 (0.38-1.79) | 0.61 (0.23-1.59) | 0.76 (0.20-2.91) | 0.99 | | 1.01 (0.78-1.30) | |  |

Multivariable model adjusted for: age, sex, ethncity, education, smoking status/time since quitting smoking/cigarettes per day, alcohol, height, body mass index, leisure-time physical activity, connective tissue disease (Marfan syndrome, Ehlers-Danlos syndrome), diabetes mellitus, low-density-lipoprotein cholesterol, lipid-lowering medications, blood pressure-lowering medications

^1^ main model

^2^ excluding persons with prevalent ischemic heart disease and stroke

^3^ excluding persons using blood pressure-lowering medications

^4^ excluding persons with prevalent ischemic heart disease and stroke and using blood pressure-lowering medications

^5^ excluding persons with prevalent ischemic heart disease and stroke and using blood pressure-lowering medications and the first 3 years of follow-up

Supplementary Table 3: Hazard ratios and 95% confidence intervals for the association between diastolic blood pressure and aortic aneurysm overall, thoracic, abdominal and unspecified aortic aneurysm, ruptured vs. non-ruptured aortic aneurysm, and aortic aneurysm mortality - sensitivity analyses excluding participants with prevalent ischemic heart disease, stroke, users of blood-pressure-lowering medications, and the first 3 years of follow-up.

| Diastolic blood pressure, cut-offs (median), mmHg | | | | | | | | |  |
| --- | --- | --- | --- | --- | --- | --- | --- | --- | --- |
| Outcome |  | <80 (74) | 80-84 (82) | 85-89 (87) | 90-99 (93.5) | 100-109 (103) | ≥110 (113) | P_trend_ | Per 10 mmHg |
|  | Participants | 206,226 | 96,709 | 81,526 | 87,506 | 20,248 | 3,327 |  |  |
|  | Person-years | 2,530,245.2 | 1,188,968.1 | 1,002,830.9 | 1,076,816.9 | 248,468.5 | 40,382.6 |  | 6,087,712 |
| Aortic aneurysm | Cases | 1,213 | 606 | 541 | 744 | 204 | 38 |  | 3,346 |
|  | HR (95% CI)^1^ | 1.00 | 0.95 (0.86-1.05) | 0.95 (0.86-1.05) | 1.19 (1.08-1.31) | 1.43 (1.23-1.67) | 1.74 (1.26-2.41) | <0.0001 | 1.08 (1.04-1.12) |
|  | HR (95% CI)^2^ | 1.00 | 0.99 (0.89-1.11) | 1.01 (0.90-1.14) | 1.25 (1.12-1.39) | 1.56 (1.32-1.84) | 1.81 (1.27-2.57) | <0.0001 | 1.12 (1.08-1.17) |
|  | HR (95% CI)^3^ | 1.00 | 1.03 (0.90-1.16) | 1.01 (0.88-1.15) | 1.31 (1.16-1.48) | 1.62 (1.34-1.96) | 1.80 (1.20-2.69) | <0.0001 | 1.13 (1.08-1.19) |
|  | HR (95% CI)^4^ | 1.00 | 1.10 (0.96-1.25) | 1.04 (0.90-1.20) | 1.34 (1.18-1.53) | 1.67 (1.37-2.04) | 1.87 (1.24-2.82) | <0.0001 | 1.16 (1.10-1.22) |
|  | HR (95% CI)^5^ | 1.00 | 1.13 (0.98-1.30) | 1.05 (0.91-1.23) | 1.38 (1.20-1.58) | 1.71 (1.40-2.11) | 1.91 (1.24-2.94) | <0.0001 | 1.17 (1.12-1.23) |
| Thoracic aortic aneurysm | Cases | 308 | 143 | 115 | 158 | 43 | 8 |  | 775 |
|  | HR (95% CI)^1^ | 1.00 | 0.85 (0.70-1.04) | 0.76 (0.61-0.94) | 0.93 (0.76-1.13) | 1.08 (0.78-1.50) | 1.30 (0.64-2.63) | 0.76 | 0.96 (0.89-1.04) |
|  | HR (95% CI)^2^ | 1.00 | 0.88 (0.71-1.09) | 0.74 (0.59-0.94) | 0.90 (0.72-1.11) | 0.94 (0.66-1.35) | 1.21 (0.57-2.58) | 0.36 | 0.94 (0.87-1.02) |
|  | HR (95% CI)^3^ | 1.00 | 0.97 (0.76-1.24) | 0.82 (0.62-1.08) | 1.16 (0.91-1.47) | 1.31 (0.89-1.94) | 1.00 (0.37-2.72) | 0.23 | 1.03 (0.94-1.13) |
|  | HR (95% CI)^4^ | 1.00 | 0.99 (0.77-1.28) | 0.81 (0.61-1.07) | 1.11 (0.86-1.42) | 1.22 (0.81-1.84) | 1.02 (0.38-2.77) | 0.46 | 1.01 (0.92-1.11) |
|  | HR (95% CI)^5^ | 1.00 | 1.05 (0.81-1.36) | 0.85 (0.63-1.14) | 1.17 (0.90-1.51) | 1.25 (0.82-1.90) | 1.13 (0.42-3.06) | 0.28 | 1.05 (0.95-.1.15) |
| Abdominal aortic aneurysm | Cases | 715 | 370 | 335 | 461 | 124 | 23 |  | 2,028 |
|  | HR (95% CI)^1^ | 1.00 | 1.01 (0.89-1.15) | 1.04 (0.91-1.18) | 1.31 (1.17-1.48) | 1.58 (1.30-1.92) | 1.95 (1.28-2.96) | <0.0001 | 1.13 (1.08-1.18) |
|  | HR (95% CI)^2^ | 1.00 | 1.08 (0.93-1.27) | 1.18 (1.01-1.39) | 1.47 (1.27-1.70) | 1.90 (1.53-2.36) | 2.18 (1.39-3.43) | <0.0001 | 1.22 (1.15-1.28) |
|  | HR (95% CI)^3^ | 1.00 | 1.05 (0.89-1.25) | 1.10 (0.92-1.31) | 1.42 (1.21-1.67) | 1.70 (1.32-2.18) | 2.17 (1.31-3.60) | <0.0001 | 1.18 (1.11-1.25) |
|  | HR (95% CI)^4^ | 1.00 | 1.14 (0.94-1.37) | 1.16 (0.96-1.41) | 1.49 (1.25-1.77) | 1.77 (1.36-2.31) | 2.25 (1.33-3.79) | <0.0001 | 1.22 (1.14-1.30) |
|  | HR (95% CI)^5^ | 1.00 | 1.15 (0.95-1.40) | 1.15 (0.94-1.42) | 1.49 (1.23-1.79) | 1.78 (1.35-2.35) | 2.15 (1.23-3.77) | <0.0001 | 1.21 (1.13-1.30) |
| Unspecified aortic aneurysm | Cases | 181 | 86 | 89 | 117 | 37 | 7 |  | 517 |
|  | HR (95% CI)^1^ | 1.00 | 0.88 (0.68-1.15) | 1.02 (0.79-1.33) | 1.21 (0.95-1.54) | 1.67 (1.16-2.40) | 2.02 (0.94-4.33) | 0.003 | 1.14 (1.04-1.24) |
|  | HR (95% CI)^2^ | 1.00 | 0.96 (0.72-1.28) | 1.08 (0.81-1.44) | 1.24 (0.95-1.63) | 1.87 (1.27-2.74) | 2.05 (0.90-4.67) | 0.001 | 1.16 (1.05-1.29) |
|  | HR (95% CI)^3^ | 1.00 | 1.08 (0.79-1.49) | 1.13 (0.82-1.57) | 1.30 (0.96-1.78) | 2.14 (1.39-3.29) | 2.34 (0.95-5.79) | 0.001 | 1.21 (1.08-1.36) |
|  | HR (95% CI)^4^ | 1.00 | 1.22 (0.87-1.70) | 1.19 (0.84-1.70) | 1.43 (1.03-1.98) | 2.46 (1.59-3.82) | 2.66 (1.07-6.60) | <0.0001 | 1.26 (1.12-1.42) |
|  | HR (95% CI)^5^ | 1.00 | 1.25 (0.88-1.78) | 1.26 (0.87-1.82) | 1.58 (1.12-2.22) | 2.70 (1.71-4.24) | 3.04 (1.22-7.58) | <0.0001 | 1.32 (1.17-1.49) |
| Ruptured aortic aneurysm | Cases | 41 | 25 | 12 | 33 | 10 | 0 |  | 121 |
|  | HR (95% CI)^1^ | 1.00 | 1.24 (0.75-2.05) | 0.69 (0.36-1.32) | 1.82 (1.14-2.92) | 2.48 (1.22-5.03) | - | 0.02 | 1.23 (1.03-1.47) |
|  | HR (95% CI)^2^ | 1.00 | 1.26 (0.71-2.24) | 0.50 (0.22-1.15) | 1.82 (1.06-3.14) | 2.04 (0.88-4.73) | - | 0.09 | 1.17 (0.95-1.45) |
|  | HR (95% CI)^3^ | 1.00 | 1.38 (0.71-2.69) | 0.94 (0.43-2.06) | 2.22 (1.20-4.11) | 3.07 (1.28-7.37) | - | 0.009 | 1.32 (1.05-1.66) |
|  | HR (95% CI)^4^ | 1.00 | 1.46 (0.74-2.89) | 0.77 (0.32-1.86) | 2.00 (1.04-3.86) | 2.23 (0.81-6.10) | - | 0.09 | 1.22 (0.95-1.57) |
|  | HR (95% CI)^5^ | 1.00 | 1.66 (0.82-3.34) | 0.50 (0.17-1.51) | 1.80 (0.88-3.70) | 2.07 (0.68-6.29) | - | 0.26 | 1.17 (0.89-1.53) |
| Non-ruptured aortic aneurysm | Cases | 1,172 | 581 | 529 | 711 | 194 | 38 |  | 3,225 |
|  | HR (95% CI)^1^ | 1.00 | 0.94 (0.85-1.04) | 0.96 (0.86-1.06) | 1.17 (1.06-1.29) | 1.40 (1.20-1.64) | 1.79 (1.29-2.47) | <0.0001 | 1.08 (1.04-1.11) |
|  | HR (95% CI)^2^ | 1.00 | 0.98 (0.88-1.11) | 1.03 (0.91-1.16) | 1.23 (1.10-1.37) | 1.54 (1.30-1.82) | 1.86 (1.31-2.64) | <0.0001 | 1.12 (1.08-1.17) |
|  | HR (95% CI)^3^ | 1.00 | 1.01 (0.89-1.15) | 1.01 (0.88-1.15) | 1.28 (1.13-1.46) | 1.58 (1.30-1.91) | 1.84 (1.23-2.75) | <0.0001 | 1.13 (1.08-1.18) |
|  | HR (95% CI)^4^ | 1.00 | 1.08 (0.94-1.24) | 1.05 (0.90-1.21) | 1.32 (1.16-1.51) | 1.65 (1.35-2.02) | 1.92 (1.27-2.90) | <0.0001 | 1.16 (1.10-1.21) |
|  | HR (95% CI)^5^ | 1.00 | 1.11 (0.96-1.28) | 1.07 (0.92-1.25) | 1.37 (1.19-1.57) | 1.70 (1.38-2.10) | 1.96 (1.27-3.02) | <0.0001 | 1.17 (1.11-1.24) |
| Aortic aneurysm mortality | Cases | 58 | 28 | 34 | 51 | 11 | 2 |  | 184 |
|  | HR (95% CI)^1^ | 1.00 | 1.01 (0.64-1.60) | 1.40 (0.91-2.16) | 1.99 (1.35-2.93) | 1.93 (1.00-3.72) | 2.32 (0.56-9.58) | <0.0001 | 1.25 (1.09-1.45) |
|  | HR (95% CI)^2^ | 1.00 | 1.14 (0.67-1.94) | 1.43 (0.85-2.41) | 2.29 (1.45-3.62) | 2.12 (1.01-4.45) | 1.51 (0.20-11.08) | 0.001 | 1.28 (1.08-1.52) |
|  | HR (95% CI)^3^ | 1.00 | 0.64 (0.31-1.32) | 1.59 (0.91-2.78) | 2.14 (1.28-3.59) | 2.67 (1.25-5.73) | 3.80 (0.90-16.15) | <0.0001 | 1.34 (1.11-1.62) |
|  | HR (95% CI)^4^ | 1.00 | 0.62 (0.28-1.38) | 1.64 (0.89-3.01) | 2.39 (1.37-4.16) | 2.82 (1.24-6.39) | 2.11 (0.28-15.77) | <0.0001 | 1.37 (1.12-1.69) |
|  | HR (95% CI)^5^ | 1.00 | 0.52 (0.21-1.29) | 1.36 (0.69-2.69) | 2.22 (1.22-4.06) | 2.75 (1.15-6.59) | - | 0.004 | 1.30 (1.03-1.64) |

Multivariable model adjusted for: age, sex, ethncity, education, smoking status/time since quitting smoking/cigarettes per day, alcohol, height, body mass index, leisure-time physical activity, connective tissue disease (Marfan syndrome, Ehlers-Danlos syndrome), diabetes mellitus, low-density-lipoprotein cholesterol, lipid-lowering medications, blood pressure-lowering medications

^1^ main model

^2^ excluding persons with prevalent ischemic heart disease and stroke

^3^ excluding persons using blood pressure-lowering medications

^4^ excluding persons with prevalent ischemic heart disease and stroke and using blood pressure-lowering medications

^5^ excluding persons with prevalent ischemic heart disease and stroke and using blood pressure-lowering medications and the first 3 years of follow-up
